# Comparison of lidocaine, huffing maneuver and combination of both in prevention fentanyl induced cough before induction of anesthesia: a double-blind, prospective, randomized placebo-controlled study

**DOI:** 10.1186/s12871-021-01313-w

**Published:** 2021-03-27

**Authors:** Laleh Dehghanpisheh, Mohammadhossein Eghbal, Fatemeh Bagheri Baravati, Pooya Vatankhah

**Affiliations:** Anesthesiology and Critical Care Research Center, Shiraz University of Medical Sciences, Shiraz, Iran

**Keywords:** Fentanyl, Lidocaine, Huffing maneuver, Cough, Combination protocol

## Abstract

**Background:**

Intravenous bolus injection of fentanyl has been frequently reported to be associated with cough reflex during patient anesthesia. However, the search for the most effective protocol continues. This study aimed to compare the effect of reducing cough reflex after injection of fentanyl in a fusion protocol by combining the injections of lidocaine and Huffing maneuver and comparing with a placebo control group, before anesthesia induction.

**Methods:**

This prospective randomized controlled trial study was performed on 400 patients who were divided into four groups of combined protocol (group 1), lidocaine group (group 2), Huffing maneuver group (group 3), and the control receiving normal saline (group 4). Then patients were injected with 2. 5 μg /kg fentanyl and monitored for 2 min regarding their cough reflex, as well as the severity.

**Results:**

In group one, 9 patients (9%), in group two, 45 patients (45%), 22 patients (22%) in group three, and in group four, 75 patients (75%), developed cough reflex following fentanyl injection. Also, 13 patients (13%) developed moderate and 4 (4%) developed severe coughs in the control group reported, while no reports of severe or moderate cough were among the intervention groups. There was a significant difference between the intervention group and the control group both in terms of the rate and severity of the fentanyl-induced cough.

**Conclusion:**

By using a combination of lidocaine injection along and Huffing maneuver, better results can be obtained in reducing the frequency, and also the severity of cough followed by fentanyl injection.

**Trial registration:**

The trial was registered with IRCT.IR (09/03/2018-No. IRCT20141009019470N74).

## Introduction

Fentanyl-induced cough (FIC) is amongst the most common complications of anesthesia induction by intravenous bolus administration of fentanyl, which has been reported in 18 to 65% of patients [1, 2]. Although this phenomenon is usually brief and self-limiting, during anesthesia induction, coughing is undesirable since it is associated with increased intracranial (ICP), intraocular, and intra-abdominal pressures. Also, multiple conjunctival and periorbital petechiae can be triggered by extreme FIC [3] and contribute to upper airway obstruction that may need urgent intervention [4]. Hence, prevention of FIC is of much clinical value.

Although multiple FIC-responsible mechanisms have been proposed, the exact mechanism remains unknown. Central sympathetic outflow could be blocked by fentanyl, thus stimulating the vagus nerve. This vagal activity change has been identified as a potential cause of cough and reflex bronchoconstriction [5, 6]; However, a study by Lui Ping-Wing et al. [5] reported that cough reflex after opioid injection is not likely to be vagally mediated because atropine pretreatment does not affect it. Hence, further pathophysiological studies in this area are required for the better understanding of the exact mechanism of FIC.

Lidocaine was successful in suppressing FIC in a meta-analysis by Kim et al., regardless of dose [7]. The mechanism by which lidocaine suppresses mechanically and chemically mediated cough reflex remains unclear, although it has been proposed that depression in brain-stem activity is a possibility [8].

The Huffing maneuver is a gentle, voluntary maneuver that opens the glottis following a high-pressure exhalation, and the air inside the upper airways is emptied along with secretions. This maneuver causes the opening of closed alveoli, reduces atelectasis, and increases the functional residual capacity of the lungs [9, 10]. Ambesh et al. reported a significant decrease in the incidence and severity of FIC in patients who perform a Huffing maneuver just before intravenous fentanyl injection [11].

Various interventions have been performed to reduce the incidence of FIC, however none of which alone have demonstrated satisfactory reducing effect. In this study, the reducing effect of FIC was investigated in an integrated protocol with the simultaneous use of lidocaine injection along with the Huffing maneuvers before induction of anesthesia. We aimed to evaluate the incidence of FIC, along with its severity after lidocaine injection, Huffing maneuver and a combination of both in order to provide guidelines for anesthesiologists worldwide.

## Material and method

This study, which is a prospective randomized controlled trial, was performed on 400 patients who underwent fentanyl injection in hospitals affiliated to Shiraz University of Medical Sciences in 2018. Patients with non-emergency surgery aged 14 to 69 years were included in the study, while patients with a history of asthma, chronic obstructive pulmonary disease, chronic cough, upper respiratory tract infection in the 2 weeks before surgery, a history of smoking, steroid or bronchodilator treatment, or people taking ACE inhibitors were excluded.

The study was approved by the ethics committee of Shiraz University of Medical Sciences (IR.SUMS.MED.REC.1394.61), the institutional review board, and Iranian Registry of Clinical Trials (IRCT.ir; date: 09/03/2018-No. IRCT20141009019470N74) and conducted in compliance with local regulatory requirements, Good Clinical Practice (GCP), and the Declaration of Helsinki [12]. All stages of the research and the goals were explained by the researcher to all the patients and written informed consent was obtained from all patients or their legally authorized representatives. Also, it is ensured that the reluctance to participate in the study has no effect on their treatment process and they can leave our study whenever they wish.

Regarding sample size estimation, assuming 95% confidence level (first type alpha error 5%) and 80% power and considering the observation of at least 0.30 and expecting differences in treatment results between the intervention (0.25) and control (0.55) groups, we calculated that a total of 164 patients (i.e. 41 cases in the intervention group and 41 in the control group) would be required for the analysis (Fleiss with CC). By applying a design effect of 2 based on our inclusion and exclusion criteria and multicentral study, reaching a total estimation of 328 patients.

The Patients were then divided into four groups of 100 which were equivalent in sex and age, based on block randomization.

Group 1 (Integrated Protocol Group): In this group, 1 min before fentanyl injection, 2 mg/kg lidocaine was rapidly (during 10 s) administered through the peripheral iv line for the patient and after 55 s, Huffing maneuver was performed after a deep inspiration for less than 5 s, and subsequently the patient was administered 2.5 μg /kg fentanyl. Huffing maneuver was performed in accordance with standard procedures [13], in which patients were advised to start with diaphragmatic breathing in every postural drainage position. This was followed by thoracic expansion exercises and diaphragmatic breathing again after the patient had relaxed sufficiently. Then two huffs followed, with chest compression alternating with relaxed diaphragmatic breathing (maximum forced expiration from mid-lung volume). The patients coughed when required. This procedure was carried out without any assistance. The act of huffing lasted < 5 s and was standardized to all patients. Regarding bolus injection of lidocaine, since slow injection of fentanyl above 30 s seems to be effective in preventing FIC, [7] bolus injection was performed during 10 s in all lidocaine groups.

Group 2 (lidocaine group): In this group, 1 min before fentanyl injection, 2 mg/kg lidocaine was rapidly (during 10 s) administered through the peripheral iv line for the patient and then fentanyl was was rapidly administered through the peripheral iv line for the patient at 2.5 μg/kg.

Group 3 (Huffing group): In this group, 1 min before fentanyl injection, the Huffing maneuver was performed after a deep inspiration for less than 5 s and then fentanyl was rapidly administered through the peripheral iv line for a rate of 2.5 μg /kg.

Group 4 (control group): In this group, 1 min before the administration of fentanyl, an equal volume of normal saline with the lidocaine group was rapidly administered through the peripheral iv line for the patient and then fentanyl was rapidly administered through the peripheral iv line at the rate of 2. 5 μg /kg.

Then the patients were monitored by a blinded research assistant for 2 min, which was blinded from the patients grouping, and the presence or absence of reflex cough as well as the severity of cough (as 1–2 coughs assigned as first-degree cough, 3–4 as grade 2, while 5 coughs and more than 5 grade 3) was reported in each patient.

During these 2 min, all secondary changes related to fentanyl such as changes in heart rate or changes in blood pressure that required intervention were recorded. For the induction of anesthesia, propofol was administered for all patients if no coughs were recorded during the initial 2 min after fentanyl injection, while in cases where coughs persisted, propofol injection was pushed forward and induction was performed. All data were collected and entered into SPSS statistical software for statistical analysis and analyzed using the chi-square test for qualitative variables and student test for quantitative variables. *P*-value < 0.05 was also considered significant.

## Results

A total of 400 patients in four groups were included in the study during 2018. Patients were matched in terms of age and sex in groups (*P* > 0.05). The baseline demographic data of the patients is demonstrated in Table 1.

**Table 1:** Demographic and Fentanyl-induced cough frequency and severity based on the administration of Lidocaine, Huffing Maneuver, or combined therapy

Variables	Groups *n = 400*				*P*-value*
	Integrated Protocol Group *n = 100*	Lidocaine group *n = 100*	Huffing group *n = 100*	Control group *n = 100*	
Age	42.67 ± 12.73	40.99 ± 10.75	44.05 ± 14.84	41.24 ± 15.03	0.345
Sex					
*Male*	45 (45%)	50 (50%)	47 (47%)	47 (47%)	0.916
*Female*	55 (55%)	50 (50%)	53 (53%)	53 (53%)	
Fentanyl-induced cough frequency	9 (9%)	45 (45%)	22 (22%)	75 (75%)	< 0.001
Severity					
*Moderate*	0 (0%)	0 (0%)	0 (0%)	13 (13%)	< 0.001
*Severe*	0 (0%)	0 (0%)	0 (0%)	4 (4%)	

* ANOVA test for age; Chi-square test for sex, and cough evaluation

As demonstrated in Table 1, the frequency of FIC in intervention groups was significantly lower than the control group (*P* value< 0.001), also this symptom was significantly lower in the first group (integrated protocol) compared to the second groups (lidocaine injection alone; *P* < 0.001) And the third (Huffing maneuver alone; *P* = 0.009). Furthermore, comparing the second and third group (lidocaine injection alone vs. Huffing maneuver alone), the rates were significantly lower in the Huffing maneuver group (*P* value< 0.001).

In the control group, 13 cases (13%) had moderate coughs and 4 cases (4%) had severe coughs, while in the intervention groups, moderate and severe cough was not observed, while this difference between the control and intervention groups was also statistically significant (*P*-value < 0.001).

## Discussion

During a cough, the pressure inside the chest reaches 300 mmHg and the expiratory air velocity reaches 500 miles per hour [14]. This pressure and speed caused by coughing can cause adverse effects such as headache, dizziness, myalgia, hoarseness, urinary incontinence, and even rib fractures, especially in patients with low bone density [15, 16]. Fentanyl is widely used in the operating room to anesthetize patients, but a typical complication of this valuable drug in patients in anesthesia induction is cough [17, 18].

In the present study, which aimed to combine the effects of Huffing maneuver and lidocaine injection before fentanyl administration to reduce the complication of FIC, it was found that FIC is prevalent after fentanyl injection in patients who no interventions in this regard was applied, which accounted for 75% of the patients in the control group. Our higher rates compared to most studies in this field, such as the study of Lin et al. [2], which reported a prevalence of 65%, and the study of Chandra et al., [19] which reported a prevalence of 34%, may have been due to the wider age range and the larger number of samples in this study.

Based on our data, the prevalence of cough was 45% in patients who received 2 μg / kg lidocaine before fentanyl injection, which was much higher compared to other studies such as Gecaj-Gashi et al. [20] who reported a prevalence of 16% with 1 μg / kg lidocaine injection and 22% with 0.5 μg / kg injection and Chandra et al. who reported a prevalence of 13% [21]. This could indicate that increasing the dose of lidocaine, as shown in another study by Chandra et al. [22], increased the prevalence of this complication. However, further studies in this regard are justified.

In our study, the incidence of cough was reported to be 22% in patients who underwent Huffing maneuvers before fentanyl injection. By applying a combination therapy, this rate was only observed in 9% of the patients, which was significantly lower compared to the control group. In this regard, a study by Ambesh et al. [11] showed that patients who underwent Huffing maneuvers before fentanyl injection had significantly lower incidences of drug-induced cough in the control group, however a study on the effect of the combination of Huffing maneuver along with lidocaine injection has not been previously reported till date. Also in the present study, similar to the study by Ambesh et al. [11], the severity of drug-induced cough in the intervention groups was significantly lower than the control group, however, in other studies, no significant relationship was observed between the groups [2, 21].

Another point of view which should be assessed is that the time of this report was during the ongoing coronavirus disease (COVID-19) pandemic, in which many countries have been affected by the disease and cough being the main transmission route of the virus [23–26]. However, the diagnosis and treatment of these patients are still not completely sufficient [27–30]. Furthermore, many patients are asymptomatic and undiagnosed and undergo various surgical procedures [30]. Aminnejad et al. [31] pointed out the value of preventing FIC to reduce the virus transmission and possible infection of healthcare workers, which is among the most damaging consequences of this pandemic among other aspects [32, 33]. As our data demonstrated, the Huffing maneuver was more effective in reducing the frequency of FIC compared to lidocaine injection. However, both techniques had significant reduction in FIC frequency compared to the control group. Therefore, by utilizing a combination approach in patients during anesthesia, remarkable reductions in FIC frequency can be obtained, as also has been demonstrated in our results.

Among the limitations and aspects that should be considered in our study is that factors such as increasing age, cigarette smoking, prior epidural injection of lidocaine or a priming dose of vecuronium have been reported to be associated with FIC, which were all excluded and matched among our groups. Therefore, evaluation of such features was not possible in our study [34–36].

## Conclusion

By using a combination of lidocaine injection along and Huffing maneuver, better results can be obtained in reducing the frequency, and also the severity of cough followed by fentanyl injection. These results can be beneficial for anesthesiologists worldwide to avoid undesirable complications of FIC.

## Data Availability

The dataset supporting the conclusions of this article is included within the article. Please contact the corresponding author in case of requiring any further information.
